# The contribution of sodium reduction and potassium increase to the blood pressure lowering observed in the Salt Substitute and Stroke Study

**DOI:** 10.1038/s41371-024-00896-4

**Published:** 2024-02-21

**Authors:** Liping Huang, Qiang Li, Jason HY Wu, Maoyi Tian, Xuejun Yin, Jie Yu, Yishu Liu, Xinyi Zhang, Yangfeng Wu, Ellie Paige, Kathy Trieu, Matti Marklund, Anthony Rodgers, Bruce Neal

**Affiliations:** 1 The George Institute for Global Health, UNSW Sydney, Sydney, NSW Australia; 2 School of Population Health, UNSW Sydney, Sydney, NSW Australia; 3 School of Public Health, Harbin Medical University, Harbin, China; 4 School of Population Medicine and Public Health, Chinese Academy of Medical Sciences and Peking Union Medical College, Beijing, China; 5 School of Public Health, Nanjing Medical University, Nanjing, China; 6 Peking University Clinical Research Institute and School of Public Health, Peking University, Beijing, China; 7 National Centre for Epidemiology and Population Health, The Australian National University, Canberra, ACT Australia; 8 Department of Epidemiology, Johns Hopkins Bloomberg School of Public Health, Baltimore, MD USA; 9 Department of of Public Health and Caring Sciences, Uppsala University, Uppsala, Sweden; 10 Imperial College London, London, UK

**Keywords:** Risk factors, Cardiovascular diseases

## Abstract

The Salt Substitute and Stroke Study (SSaSS) demonstrated significant reductions in systolic blood pressure (SBP), and the risk of stroke, major cardiovascular events and total mortality with the use of potassium-enriched salt. The contribution of sodium reduction versus potassium increase to these effects is unknown. We identified four different data sources describing the association between sodium reduction, potassium supplementation and change in SBP. We then fitted a series of models to estimate the SBP reductions expected for the differences in sodium and potassium intake in SSaSS, derived from 24-h urine collections. The proportions of the SBP reduction separately attributable to sodium reduction and potassium supplementation were calculated. The observed SBP reduction in SSaSS was −3.3 mmHg with a corresponding mean 15.2 mmol reduction in 24-h sodium excretion and a mean 20.6 mmol increase in 24-h potassium excretion. Assuming 90% of dietary sodium intake and 70% of dietary potassium intake were excreted through urine, the models projected falls in SBP of between −1.67 (95% confidence interval: −4.06 to +0.73) mmHg and −5.33 (95% confidence interval: −8.58 to −2.08) mmHg. The estimated proportional contribution of sodium reduction to the SBP fall ranged between 12 and 39% for the different models fitted. Sensitivity analyses assuming different proportional urinary excretion of dietary sodium and potassium intake showed similar results. In every model, the majority of the SBP lowering effect in SSaSS was estimated to be attributable to the increase in dietary potassium rather than the fall in dietary sodium.

## Introduction

Excess dietary sodium consumption and inadequate dietary potassium consumption are both associated with higher levels of blood pressure [1]. Randomized trials that reduced dietary sodium have clearly demonstrated blood pressure-lowering effects [2], as have trials that supplemented potassium intake [3]. Potassium-enriched salt that replaces a proportion of the sodium chloride in regular salt with potassium chloride is an effective and practical approach to blood pressure-lowering that combines these effects [4].

A recent large-scale randomized trial, the Salt Substitute and Stroke Study (SSaSS) [5], involving 20 995 participants in rural China, demonstrated significant reductions in the risk of stroke (14%, *p* = 0.006), major cardiovascular events (13%, *p* < 0.001) and premature death (12%, *p* < 0.001) with potassium-enriched salt compared to regular salt over five years [5]. The design of SSaSS was premised on the joint blood pressure-lowering effects of sodium reduction and potassium supplementation. A minimum 3.0 mmHg lowering of systolic blood pressure with potassium-enriched salt was assumed for the trial power calculation, which was close to the 3.3 mmHg mean difference observed in the trial. The corresponding effect size on clinical outcomes was in line with the magnitude of systolic blood pressure reduction based on a previous dose-response meta-analysis of systolic blood pressure lowering and risk reduction of cardiovascular diseases [6]. This implies the effect on clinical outcomes of potassium-enriched salt was mediated through blood pressure lowering.

Assessed by 24-h urinary excretion, the mean intake of sodium was 187 mmol per day and the mean intake of potassium was 36 mmol per day at baseline [7]. The former is markedly higher than the World Health Organization’s daily recommended intake of 87 mmol [8] and the latter is markedly lower than the World Health Organization’s daily recommended intake of 90 mmol [9]. About 90% of dietary sodium [10] and a more variable percent (63–92%) of dietary potassium is excreted in the urine [11]. During the trial, there was a mean reduction of 15.2 mmol for 24-h urinary sodium excretion, a mean increase of 20.6 mmol for 24-h urinary potassium excretion and a mean decrease of 3.3 mmHg for systolic blood pressure in the potassium-enriched salt group compared to the regular salt group [5].

The effects of sodium reduction and potassium supplementation on systolic blood pressure and, therefore, the clinical outcomes in SSaSS were expected to be additive but may be interdependent, with several studies suggesting interactions [3, 12]. There has been considerable discussion regarding the likely contribution of sodium reduction versus potassium supplementation to the benefits observed in SSaSS [13]. The aim of this study was to estimate the relative contribution of changes in sodium and potassium observed in the SSaSS trial to the reduction in systolic blood pressure and, by inference, the main study outcomes.

## Materials and methods

Briefly, we used the previously reported mean between-group differences in 24-h urinary sodium excretion and 24-h urinary potassium excretion in the trial as a starting point. In SSaSS, urinary electrolytes were measured on a sample of participants at baseline and at each year of follow-up (sample size varied from 544 to 1986 participants). Differences for each year were estimated using analysis of covariance adjusted for clustering as a random intercept. Overall differences for the entire study were estimated using a fixed effect inverse-variance-weighted meta-analysis to pool the differences estimated at each year of follow-up [5].

**Fig. 1:**
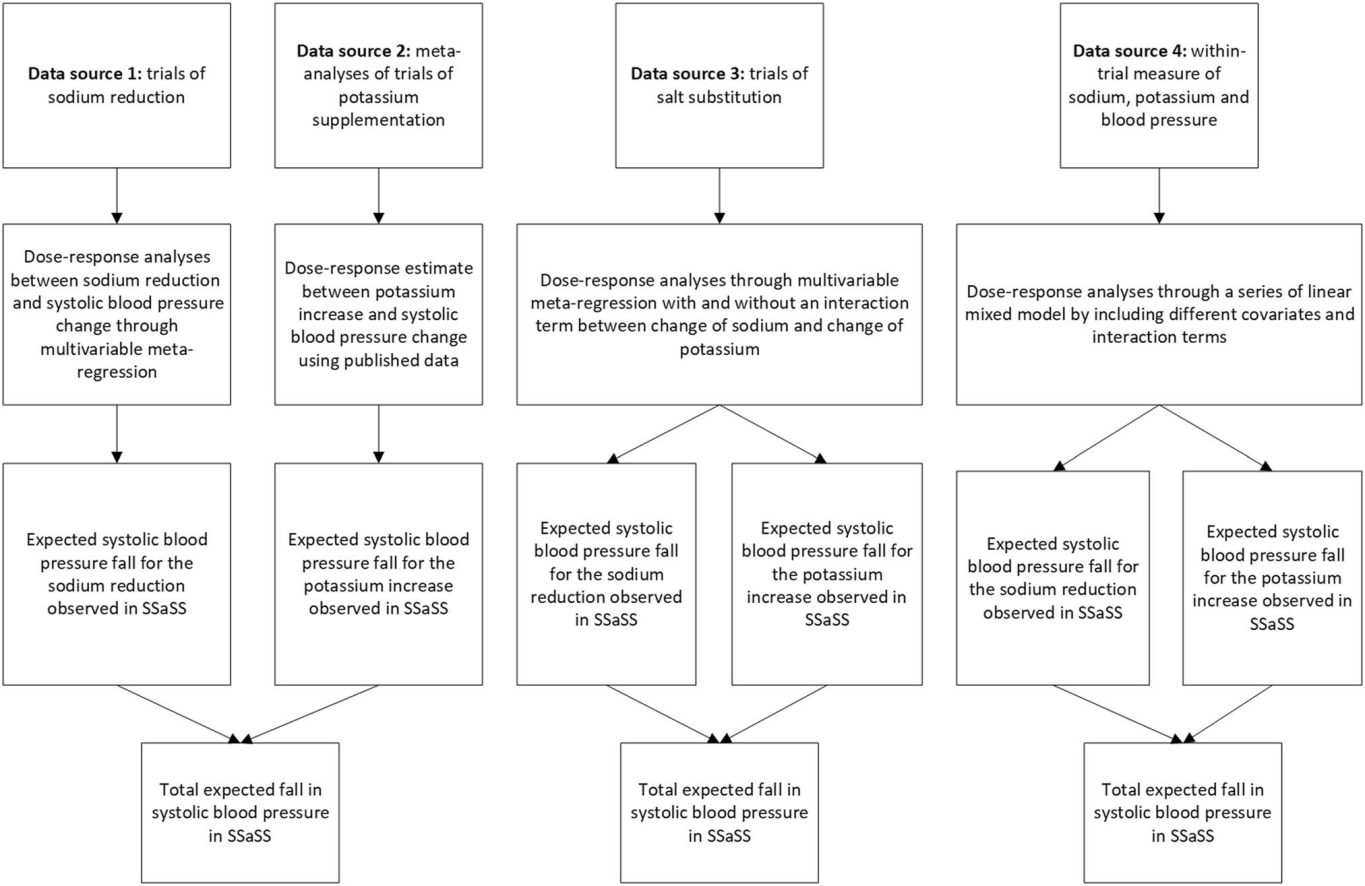
Flowchart illustrating data sources and main analytic approaches.

In this study, we employed multiple data sources describing the dose-response relationship between sodium reduction and systolic blood pressure change and between potassium increase and systolic blood pressure change to estimate the magnitude of systolic blood pressure reduction that might be expected for the observed changes in urinary sodium and potassium in SSaSS (Fig. 1). Estimates were made under varying assumptions and compared to the observed change in systolic blood pressure reported in the trial.

### Data sources for estimating the dose-response relationship between sodium, potassium and systolic blood pressure

We used four different data sources to quantify the dose-response relationships between changes in sodium, potassium and systolic blood pressure.

- *Data source 1: Randomized trials of sodium reduction* – we extracted data from our previously published systematic review and meta-analysis that quantified the dose-response relationship between sodium reduction and blood pressure [2]. We used data from the 55/133 studies with a follow-up duration of four weeks or longer (Supplementary Appendix 1). Seventy-eight studies of shorter than 4 weeks duration were not included because prior investigation indicates that the acute effects of sodium reduction on blood pressure may be different to the effects achieved in the longer term [14].
- *Data source 2: Meta-analysis of randomized trials of potassium supplementation* - we extracted published results from a systematic review and meta-analysis that quantified the relationship between potassium supplementation and blood pressure [15]. In this meta-analysis, only studies that were at least 4 weeks long were included, and other study inclusion criteria were broadly similar to the inclusion criteria for the sodium reduction trials included in data source 1. The potassium supplementation meta-analysis identified an overall non-linear relationship between potassium supplementation and blood pressure fall. The maximum systolic blood pressure fall of 3.3 mmHg (95% CI: 1.6 to 4.9) was achieved with potassium supplementation of about 30 mmol/day. Up to this level of supplementation and at the mean level of suppplementation achieved in SSaSS (i.e. 20.64 mmol/day), there was a linear association between potassium supplementation and blood pressure fall.
- *Data source 3: Randomized trials of salt substitution* - we extracted data from a recent systematic review and meta-analysis that quantified the effects of salt substitution on blood pressure [16]. We used data from the 9/21 studies of at least four weeks duration that reported data on 24-h sodium, 24-h potassium and systolic blood pressure (Supplementary Appendix 2). Eleven trials did not report the required data and therefore were not included. Also data from the SSaSS study were not included.
- *Data source 4: Within-trial measures of sodium, potassium and blood pressure* - we used data from the subset of 449 SSaSS participants who had both baseline and follow-up measurements of 24-h urinary sodium, 24-h urinary potassium and systolic blood pressure (Supplementary Appendix 3).

### Statistical analyses

We first obtained estimates of the coefficients describing the dose-response relationships between sodium reduction, potassium increase and systolic blood pressure for each data source. The methods used for each were:

- *Data source 1: Randomized trials of sodium reduction* – a multivariable meta-regression including adjustment for age, sex, ethnicity and baseline systolic blood pressure in the model was fitted.
- *Data source 2: Meta-analysis of randomized trials of potassium supplementation –* we directly extracted from the published report the result of the 1-stage natural cubic spline regression analysis that incorporated multiple levels of exposure [15].
- *Data source 3: Randomized trials of salt substitution* - we estimated the dose-response relationship between sodium reduction, potassium increase, and systolic blood pressure fall using meta-regression, with and without allowance for an interaction between the effects of sodium reduction and potassium supplementation. No adjustment for covariates was made due to the limited number of trials available and the intercept of the meta-regressions was set to zero.
- *Data source 4: Within-trial measures of sodium, potassium and blood pressure* - we estimated the dose-response relationship between sodium reduction and systolic blood pressure change as well as the dose-response relationship between potassium increase and systolic blood pressure change using a series of linear mixed models. Models included adjustments for selected covariates and interaction terms (Supplementary Appendix 4).

We next estimated the expected reductions in systolic blood pressure for the changes in urinary sodium and potassium excretions observed in SSaSS using the coefficients derived from the analyses above. This was done by multiplying the mean between-group difference in 24-h urinary sodium excretion observed in the SSaSS trial (−15.2 mmol) by the dose-response coefficient (mmHg/mmol) estimated from each different data source and statistical model. 95% CIs for the expected mean change in systolic blood pressure attributable to sodium reduction were estimated using the 95% CIs of the dose-response coefficient from each data source and statistical model. The same approach was used to calculate the expected mean reductions and corresponding 95% CIs in systolic blood pressure change attributable to the between-group differences in 24-h potassium excretion observed in SSaSS (20.6 mmol). The primary analyses were also adjusted to account for the incomplete urinary excretion of sodium (90%) [10] and potassium (70%, acknowledging there was a large variability in the proportional excretion [11]) such that the blood pressure reductions attributed to each reflected changes in dietary intake. We also did sensitivity analyses that varied the assumed proportional excretions of sodium and potassium as well as the effects based solely on the observed changes in urinary excretion.

The total expected mean change in systolic blood pressure was estimated by summing the separate estimated effects of sodium reduction and potassium increase. For data sources 1 & 2, the 95% CI about the total expected blood pressure reduction was estimated using Monte Carlo simulation where 1000 random draws of potential sodium and potassium effects on systolic blood pressure were summed and the 95% CI of the summed effect was defined by the 2.5th and 97.5th percentiles of the *n* = 1000 sums. For data sources 3 & 4, the 95% CIs about the total expected blood pressure reduction was estimated using the Delta method [17].

The proportions of systolic blood pressure reduction attributable to sodium reduction versus potassium supplementation were obtained by dividing the systolic blood pressure reduction expected for each by the total expected systolic blood pressure reduction. The estimates derived from each data source and statistical model were plotted in a stacked bar chart for visual comparison against each other.

All analyses were done using R version 4.2.2 and Rstudio version 2022.12.0 Build 353 [18]. Packages “meta” [19], “lme4” [20] and “gtsummary” [21] were used for statistical analysis and summarizing results.

## Results

### Associations between sodium reduction, potassium increase and systolic blood pressure from different data sources and statistical models

There were more than tenfold differences in the estimated coefficients of the association between sodium reduction and systolic blood pressure reduction across the different data sources and statistical models assessed (Table 1). The highest coefficient was observed in the meta-regression of trials of salt substitutes with a model that included an interaction term between the effect of sodium reduction and potassium supplementation on systolic blood pressure. In that analysis, each mmol sodium reduction was associated with a 0.136 (95% CI: 0.029 to 0.243) mmHg systolic blood pressure fall. By contrast, the lowest coefficient was a 0.012 (95% CI: −0.009 to 0.033) mmHg systolic blood pressure fall for each mmol sodium reduction observed in the analyses of the SSaSS trial participants where the model included change in sodium, change in potassium, sex, age and baseline systolic blood pressure.

**Table 1:** Associations of change in sodium (mmol) with systolic blood pressure (mmHg) estimated from different data sources and models, and the corresponding expected effect on systolic blood pressure (SBP, mmHg) in the Salt Substitute and Stroke Study (SSaSS).

Data source and analysis	No. of studies/Participants	Covariates	Association of 1 mmol reduction in urinary sodium with change in SBP (mmHg)	Expected effect on SBP (mmHg) in SSaSS trial with no adjustment to dietary intake^1^	Expected effect on SBP (mmHg) in SSaSS trial with adjustment to dietary intake^2^	Expected effect on SBP (mmHg) in SSaSS trial with adjustment to dietary intake^3^
			Estimate (95% CI)	Estimate (95% CI)	Estimate (95% CI)	Estimate (95% CI)
Data source 1: Meta-regression of trials of sodium reduction	Trials *n* = 55	Change of sodium; Sex; Age; Ethnicity; Baseline systolic blood pressure	−0.041 (−0.065 to −0.018)	−0.63 (−0.99 to −0.27)	−0.66 (−1.04 to −0.28)	−0.70 (−1.10 to −0.30)
Data source 3: Meta-regression of trials of salt substitution (with interaction)	Trials *n* = 9	Change of sodium; Change of potassium; Interaction of change of sodium and potassium	−0.136 (−0.243 to −0.029)	−2.07 (−3.70 to −0.44)	−2.18 (−3.89 to −0.46)	−2.30 (−4.11 to −0.49)
Data source 3: Meta-regression of trials of salt substitution (no interaction)	Trials *n* = 9	Change of sodium; Change of potassium	−0.121 (−0.181 to −0.060)	−1.83 (−2.75 to −0.91)	−1.93 (−2.90 to −0.96)	−2.04 (−3.06 to −1.02)
Data source 4: In trial data unadjusted (model 1)	Participants *n* = 449	Change of sodium; Change of potassium; Cluster as a random intercept	−0.021 (−0.047 to +0.005)	−0.32 (−0.72 to +0.07)	−0.34 (−0.76 to +0.08)	−0.36 (−0.80 to +0.08)
Data source 4: In trial data minimally adjusted (model 2)	Participants *n* = 449	Change of sodium; Change of potassium; Sex; Age; Baseline systolic blood pressure; Cluster as a random intercept	−0.012 (−0.033 to +0.009)	−0.19 (−0.51 to +0.13)	−0.20 (−0.53 to +0.14)	−0.21 (−0.56 to +0.15)
Data source 4: In trial data fully adjusted (model 3)	Participants *n* = 449	Change of sodium; Change of potassium; Sex; Age; Baseline systolic blood pressure; Baseline sodium; Baseline potassium; Cluster as a random intercept	−0.050 (−0.078 to −0.022)	−0.76 (−1.18 to −0.34)	−0.80 (−1.24 to −0.36)	−0.85 (−1.31 to −0.38)
Data source 4: In trial data fully adjusted with single interaction term (model 4)	Participants *n* = 449	Change of sodium; Change of potassium; Sex; Age; Baseline systolic blood pressure; Baseline sodium; Baseline potassium; Interaction between change of sodium and change of potassium; Cluster as a random intercept	−0.036 (−0.067 to −0.005)	−0.55 (−1.01 to −0.08)	−0.57 (−1.07 to −0.08)	−0.61 (−1.13 to −0.09)
Data source 4: In trial data fully adjusted with dual interaction terms (model 5)	Participants *n* = 449	Change of sodium; Change of potassium; Sex; Age; Baseline systolic blood pressure; Baseline sodium; Baseline potassium; Interaction between the change of sodium and baseline potassium; Interaction between the change of potassium and baseline sodium; Cluster as a random intercept	−0.104 (−0.160 to −0.048)	−1.58 (−2.43 to −0.73)	−1.66 (−2.56 to −0.76)	−1.75 (−2.70 to −0.81)

^1^Assuming the urinary sodium difference is equivalent to the dietary sodium intake difference.

^2^Assuming the urinary sodium difference represents 95% of the dietary sodium intake difference.

^3^Assuming the urinary sodium difference represents 90% of the dietary sodium intake difference.

For the association between potassium increase and systolic blood pressure reduction, the estimated coefficient of the associations varied by about 2.5-fold across the different data sources (Table 2). The highest coefficient was also observed in the meta-regression of trials of salt substitutes with a model that included an interaction term between the effect of sodium reduction and potassium supplementation on systolic blood pressure. In that analysis, each mmol higher potassium supplementation was associated with a 0.124 (95% CI: −0.033 to 0.281) mmHg lower systolic blood pressure. Likewise, the lowest coefficient was a 0.050 (95% CI: −0.027 to 0.126) mmHg lower systolic blood pressure for each mmol higher potassium increase observed in the analyses of the SSaSS trial participants where the model included change in sodium, change in potassium, sex, age and baseline systolic blood pressure.

**Table 2:** Associations of change of potassium (mmol) with systolic blood pressure (mmHg) estimated from different data sources and models, and the corresponding expected effect on systolic blood pressure (SBP, mmHg) in the Salt Substitute and Stroke Study (SSaSS).

Data source and analysis	No. of studies/Participants	Covariates	Association of 1 mmol increase in urinary potassium with change in SBP (mmHg)	Expected effect on SBP (mmHg) in SSaSS trial with no adjustment to dietary intake^1^	Expected effect on SBP (mmHg) in SSaSS trial with adjustment to dietary intake^2^	Expected effect on SBP (mmHg) in SSaSS trial with adjustment to dietary intake^3^
			Estimate (95% CI)	Estimate (95% CI)	Estimate (95% CI)	Estimate (95% CI)
Data source 2: “1-stage” natural cubic spline regression of randomized trials of potassium supplementation	Trials *n* = 32	N/A. Not reported.	−0.110 (−0.163 to −0.053)	−2.27 (−3.37 to −1.10)	−2.84 (−4.21 to −1.38)	−3.24 (−4.82 to −1.57)
Data source 3: Meta-regression of trials of salt substitution (with interaction)	Trials *n* = 9	Change of sodium; Change of potassium; Interaction of change of sodium and potassium	−0.124 (−0.281 to +0.033)	−2.55 (−5.79 to +0.69)	−3.19 (−7.24 to +0.86)	−3.64 (−8.27 to +0.98)
Data source 3: Meta-regression of trials of salt substitution (no interaction)	Trials *n* = 9	Change of sodium; Change of potassium	−0.112 (−0.246 to +0.023)	−2.31 (−5.08 to +0.47)	−2.88 (−6.35 to +0.59)	−3.30 (−7.26 to +0.67)
Data source 4: In trial data unadjusted (model 1)	Participants *n* = 449	Change of sodium; Change of potassium; Cluster as a random intercept	−0.057 (−0.151 to +0.038)	−1.17 (−3.12 to +0.78)	−1.46 (−3.90 to +0.97)	−1.67 (−4.45 to +1.11)
Data source 4: In trial data minimally adjusted (model 2)	Participants *n* = 449	Change of sodium; Change of potassium; Sex; Age; Baseline systolic blood pressure; Cluster as a random intercept	−0.050 (−0.126 to +0.027)	−1.02 (−2.59 to +0.55)	−1.28 (−3.24 to +0.68)	−1.46 (−3.70 to +0.78)
Data source 4: In trial data fully adjusted (model 3)	Participants *n* = 449	Change of sodium; Change of potassium; Sex; Age; Baseline systolic blood pressure; Baseline sodium; Baseline potassium; Cluster as a random intercept	−0.063 (−0.142 to +0.017)	−1.3 (−2.93 to +0.34)	−1.62 (−3.67 to +0.43)	−1.85 (−4.19 to +0.49)
Data source 4: In trial data fully adjusted with single interaction term (model 4)	Participants *n* = 449	Change of sodium; Change of potassium; Sex; Age; Baseline systolic blood pressure; Baseline sodium; Baseline potassium; Interaction between change of sodium and change of potassium; Cluster as a random intercept	−0.054 (−0.134 to +0.025)	−1.12 (−2.76 to +0.52)	−1.40 (−3.45 to +0.65)	−1.60 (−3.94 to +0.74)
Data source 4: In trial data fully adjusted with dual interaction terms (model 5)	Participants *n* = 449	Change of sodium; Change of potassium; Sex; Age; Baseline systolic blood pressure; Baseline sodium; Baseline potassium; Interaction between the change of sodium and baseline potassium; Interaction between the change of potassium and baseline sodium; Cluster as a random intercept	−0.114 (−0.310 to +0.083)	−2.34 (−6.40 to +1.72)	−2.93 (−8.01 to +2.15)	−3.35 (−9.15 to +2.46)

^1^Assuming the urinary sodium difference is equivalent to the dietary potassium intake difference.

^2^Assuming the urinary sodium difference represents 80% of the dietary potassium intake difference.

^3^Assuming the urinary sodium difference represents 70% of the dietary potassium intake difference.

### Expected effects of dietary sodium reduction and dietary potassium increase on systolic blood pressure fall in the SSaSS based on different data sources and statistical models

**Fig. 2:**
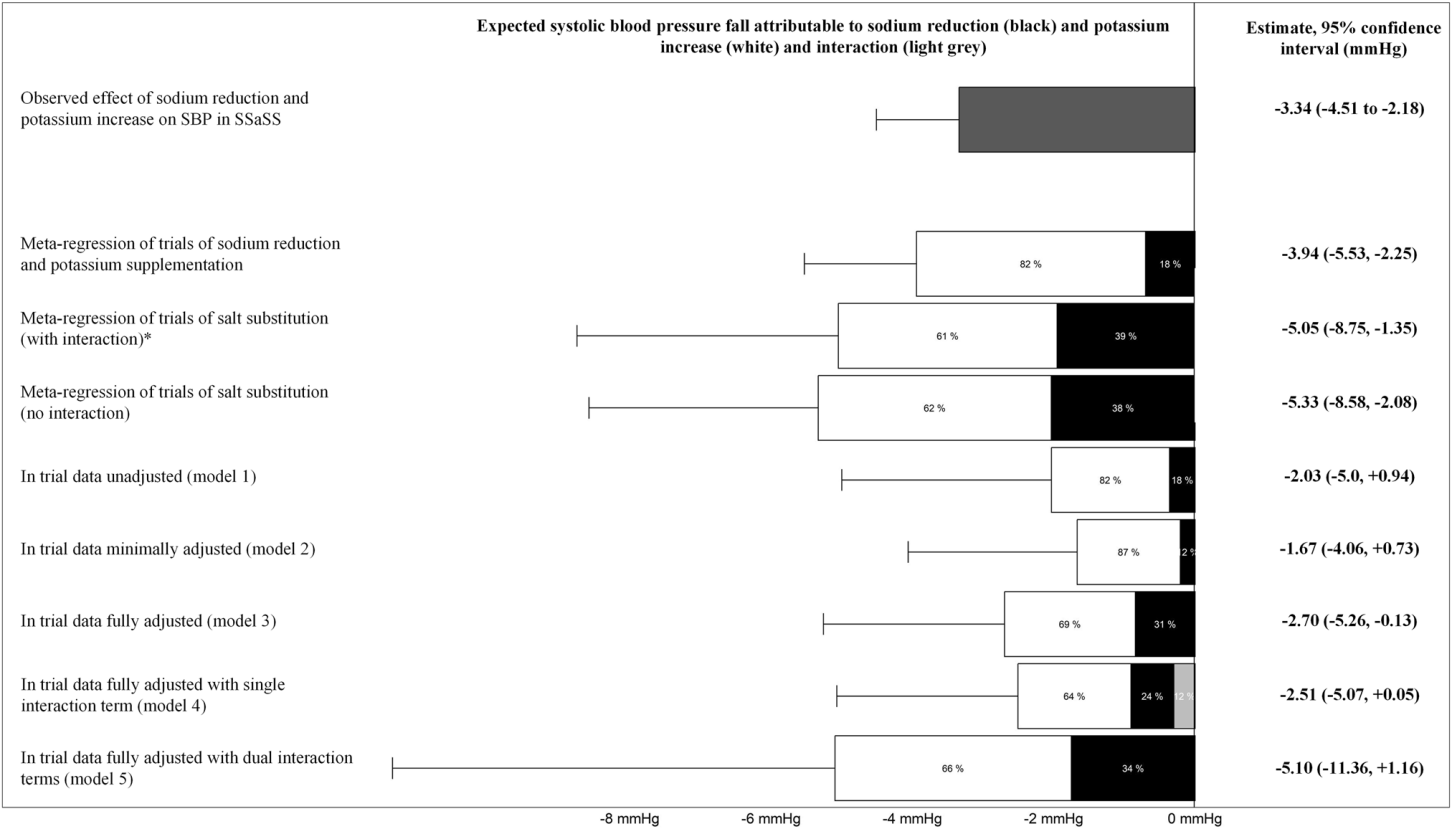
Observed systolic blood pressure reduction in the Salt Substitute and Stoke Study (dark grey) and expected effects of sodium reduction (black) and potassium increase (white) on systolic blood pressure for different data sources and statistical models assuming 90% of dietary sodium and 70% of dietary potassium were excreted through urine. * for this model, the interaction term was an 0.57 mmHg rise in SBP attributable to the joint effects of sodium reduction and potassium supplementation, which is not shown in the figure.

In the primary analyses that assumed 90% of urinary excretion of dietary sodium and 70% of urinary excretion of dietary potassium, the expected effects of sodium reduction and potassium supplementation on blood pressure in SSaSS varied between −1.67 (95% CI: −4.06 to +0.73) to −5.33 (95% CI: −8.58 to −2.08) mmHg for the different data sources and statistical models (Fig. 2). The former estimate was obtained based upon the parameters derived from the analyses of the SSaSS participants’ data where the model included change in sodium, change in potassium, sex, age and baseline systolic blood pressure, while the latter was derived from the analyses of the trials of salt substitutes that didn’t include an interaction term between the effect of sodium reduction and potassium supplementation on blood pressure. The estimated proportional contributions of sodium reduction to the fall in systolic blood pressure ranged between 12 and 39%, with the majority of the blood pressure lowering effect attributable to dietary potassium supplementation in every scenario.

In sensitivity analyses that instead assumed proportional urinary excretion of dietary sodium was 95% and proportional urinary excretion of dietary potassium was 80%, the expected systolic blood pressure reductions ranged from −1.47 (95% CI: −3.58 to +0.63) to −4.81 (95% CI: −7.65 to −1.97) mmHg. The estimated contributions of sodium reduction to the changes in blood pressure identified in these analyses ranged from 13 to 41% (Supplementary Fig. 1). In analyses that did not adjust for the incomplete urinary excretion of electrolytes, the expected systolic blood pressure reductions ranged between −1.21 (95% CI: −2.92 to +0.51) and −4.14 (95% CI: −6.41 to −1.87) mmHg and contributions of sodium reduction to these falls ranged from 15 to 45% (Supplementary Fig. 2).

## Discussion

The systolic blood pressure reduction observed in SSaSS appears to have been driven primarily by the increase in dietary potassium achieved with the potassium-enriched salt. Since cardiovascular risks are strongly associated with blood pressure levels [5], it follows that the cardiovascular health gains observed in SSaSS are also primarily attributable to potassium supplementation rather than sodium reduction. While all the analyses were imprecise and provided a range of estimates, it appears most likely that about three-quarters of the SSaSS effects derive from potassium supplementation and one-quarter from sodium reduction. The greater attribution of effects on blood pressure and clinical outcomes to potassium increase versus sodium reduction in SSaSS is not surprising given the smaller absolute and proportional effects that the potassium-enriched salt had on urinary sodium levels compared to urinary potassium levels. This meant that participants with high baseline levels of sodium intake had consumption reduced only moderately, while the very low baseline levels of potassium intake were raised substantially.

The absolute magnitude of the blood pressure reduction, as well as the proportion of the blood pressure reduction attributable to potassium supplementation versus sodium reduction, varied considerably depending on the data source and statistical model employed. The prior trials of salt substitutes were the most directly comparable external data source, and analyses based on these trials suggested the greatest contribution of sodium reduction to the effects observed. However, these analyses also moderately overestimated the expected blood pressure reduction compared to that observed in the trial and the uncertainty about the estimates was large because the meta-regressions from which the parameters were obtained were based on a small number of studies. The within-trial analyses using study participants were highly variable in the estimates provided, which likely reflects both the small dataset available and the relatively large number of models it was possible to fit.

There is some rationale for preferring models that allow for interactions between the effects on blood pressure of changing sodium and potassium, and a specific challenge with the estimates based on the coefficients derived from the datasets that assessed only sodium or potassium was the inability to incorporate the potential for interactions. Of note in this regard is the observation that the association of potassium supplementation with change in blood pressure was similar whether done alone or together with sodium reduction in the prior salt substitute trials, as was observed in this analysis (Table 2). By contrast, the association of sodium reduction with change in blood pressure was weak when done alone but much stronger when combined with potassium supplementation, as was done in the prior trials of salt substitute (Table 1). A potassium-induced natriuresis secondary to potassium supplementation [22–25] has been postulated as a physiological basis for an interaction of sodium reduction and potassium excretion on blood pressure.

The key strength of this analysis is the use of multiple different data sources and a range of statistical models to estimate the likely effects of sodium reduction versus potassium supplementation on systolic blood pressure, and by inference, cardiovascular outcomes. While the precision of the estimates is limited in every case, the point estimates of effect from all approaches point to a greater contribution from potassium supplementation than sodium reduction. This coherence provides some reassurance about the likely validity of the main conclusions. The analyses were based on constant urinary sodium and potassium excretion proportional to dietary intake, while the excretion of the two electrolytes may be interdependent. Because the analyses of the prior trials were all based on summary data, there was limited capacity to explore possible interactions between sodium reduction, potassium increase, each other and participant characteristics. This introduces an element of uncertainty in our conclusions about the relative contributions of sodium reduction and potassium increase to the effects observed that we were not able to address. An exploration of the prior trials of salt substitutes based upon individual participant data might be helpful in clarifying how changes to sodium and potassium interact to modify blood pressure and the extent to which these effects are modified by baseline levels of sodium and potassium intake. Also studies testing the effects of salt substitutes with different proportions of sodium and potassium, at different background intake levels of sodium and potassium would provide additional insight into how the effects of sodium reduction and potassium supplementation will influence blood pressure in different settings.

This analysis of the relative contributions of sodium reduction versus potassium supplementation to the effects observed in SSaSS provides new insight into the likely central role of potassium in the mechanism underpinning the observed clinical benefits in the trial. The findings also have implications for the implementation of the SSaSS findings, with potassium-enriched salt use likely to be most effective for disease prevention in communities where average potassium intake is low. While the effect of potassium supplementation from salt substitute may not be as effective in communities with higher baseline potassium intake such as the USA, the data also suggest that efforts to increase potassium levels in processed foods would be a worthwhile goal in parallel with sodium reduction programs. In practice, many ingredients in packaged foods start with high levels of potassium, which are heavily diluted during processing [26]. Finally, efficient targeting of public health campaigns seeking to maximize the benefits of switching the salt supply to potassium-enriched salt will require robust data describing current levels of both sodium and potassium intake in communities all around the world.

## Summary

### What is known about the topic

- Reducing sodium intake and increasing potassium intake both lower blood pressure.
- Sodium and potassium may interact with each other in their effect on blood pressure. Therefore, when sodium reduction and potassium supplementation are implemented together, the relative contribution of each is of interest.

### What this study adds

- In The Salt Substitute and Stroke Study, potassium supplementation drove about three-quarters or more towards the blood pressure-lowering effect, highlighting the importance of potassium supplementation.
- The results suggest that potassium supplementation is an important strategy for dietary prevention of cardiovascular disease in parallel to sodium reduction.

### Supplementary information

Supplementary materials

## Data Availability

All data used in the analysis have been included in this manuscript in the supplementary materials except individual data from the Salt Substitute and Stroke Study. According to China’s Personal Information Protection Law, all data generated in China cannot be shared without individual consent from the study participants. Therefore, we will not be able to share the individual data from the Salt Substitute and Stroke Study.

## Code availability

Codes used to generate the results can be made available upon request.
