# Supplementary material for: The contribution of sodium reduction and potassium increase to the blood pressure lowering observed in the Salt Substitute and Stroke Study
Source: J Hum Hypertens. 2024 Feb 21;38(4):298–306. doi: 10.1038/s41371-024-00896-4 (PMC11001572; doi:10.1038/s41371-024-00896-4)
Supplement: Supplementary file 1 — Supplementary materials [file 41371_2024_896_MOESM1_ESM.docx]

# Supplementary Appendix 1 Data of sodium reduction trials

| **Study** | **Study country** | **No. of participants** | **Mean (range) age (years)** | **Female (%)** | **White (%)** | **Baseline SBP** | **Design** | **Study duration (days)** | **Change in UNa (mmol/24-hour)** | **Difference in change of BP (mmHg)** | |
| --- | --- | --- | --- | --- | --- | --- | --- | --- | --- | --- | --- |
|  |  |  |  |  |  |  |  |  |  | **Systolic (SE)** | **Diastolic (SE)** |
| Parijs 1973^1^ | Belgium | 15 | 41 | 55 | NR | 175 | X | 28 | -98 | -6.7 (3.48) | 3.2 (4.24) |
| MacGregor 1982^2^ | UK | 19 | 49 (30-66) | 26 | 63 | 154 | X | 28 | -76 | -10.0 (2.40) | -5.0 (1.47) |
| Puska 1983^3^ | Russia | 72 | NR | NR | 100 | 153 | P | 42 | -117 | 0.1 (3.23) | -0.7 (2.29) |
| Silman 1983^4^ | UK | 25 | NR | NR | NR | 155 | P | 365 | -53 | -8.7 (10.22) | -6.3 (4.42) |
| Watt 1983^5^ | UK | 18 | 52 (31-64) | 67 | 100 | 137 | X | 28 | -56 | -0.5 (1.50) | -0.3 (0.80) |
| Erwteman 1984^6^ | Netherlands | 94 | 46 | 38 | 76 | 157 | P | 168 | -58 | -2.7 (2.20) | -3.4 (1.70) |
| Gillies 1984^7^ | Australia | 24 | 57 | 42 | NR | 147 | X | 42 | -77 | -2.4 (3.63) | -2.6 (2.46) |
| Richards 1984^8^ | New Zealand | 12 | NR | 33 | 100 | 150 | X | 28 | -105 | -5.2 (4.10) | -1.8 (3.55) |
| Grobbee 1987^9^ | Netherlands | 40 | 24 | 15 | NR | 137 | X | 42 | -72 | -0.9 (1.80) | 0.2 (1.67) |
| MacGregor 1987^10^ | UK | 15 | 52 (33-71) | 27 | 67 | 150 | X | 30 | -100 | -13.0 (3.14) | -9.0 (3.02) |
| Morgan 1987^11^ | Australia | 20 | 60.5 (50-65) | 0 | NR | 143 | P | 60 | -62 | -6.0 (5.00) | -4.2 (2.95) |
| Nowson 1988^12^ | Australia | 107 | 52 | 15 | 100 | 150 | P | 84 | -47 | -5.1 (1.42) | -4.2 (0.85) |
| Nowson 1988^12^ | Australia | 105 | 52 | 15 | 100 | 149 | P | 84 | -51 | -0.2 (1.42) | 0.5 (0.99) |
| Staessen 1988^13^ | Belgium | 1510 | 41 | 48 | NR | 131 | P | 1825 | -12 | -0.2 (1.73) | -1.0 (1.09) |
| Chalmers 1989^14^ | Australia | 88 | 59 | 17 | 100 | 152 | X | 56 | -67 | -3.6 (0.70) | -2.1 (0.40) |
| Chalmers 1989^15^ | Australia | 108 | 59 | 17 | 100 | 144 | P | 56 | -71 | -5.5 (1.48) | -2.8 (0.85) |
| Dodson 1989^16^ | UK | 9 | 62 | 33 | NR | 171 | X | 30 | -76 | -9.7 (5.80) | -5.1 (2.94) |
| MacGregor 1989^17^ | UK | 20 | 57 (42-72) | 45 | 75 | 163 | X | 30 | -141 | -16.0 (4.12) | -9.0 (2.32) |
| Parker 1990^18^ | Australia | 59 | 52 | 0 | NR | 138 | P | 28 | -93 | 1.0 (1.90) | 0.4 (1.10) |
| Carney 1991^19^ | Australia | 11 | 54 (30-65) | 55 | NR | 144 | X | 42 | -102 | -1.0 (5.57) | 1.0 (3.61) |
| Singer 1991^20^ | UK | 21 | 54 | 38 | 71 | 147 | X | 30 | -91 | -9.0 (2.34) | -3.0 (1.05) |
| Alli 1992^21^ | Italy | 56 | 48 | 57 | NR | 149 | P | 365 | 8 | -6.3 (3.06) | -3.8 (1.32) |
| Arroll 1992^22^ | New Zealand | 181 | 55 | 48 | NR | 145 | P | 180 | -1 | -0.5 (2.50) | 1.5 (1.50) |
| Benetos 1992^23^ | France | 20 | 42 | 55 | 100 | 149 | X | 28 | -78 | -6.5 (1.67) | -3.7 (1.29) |
| Cobiac 1992^24^ | Australia | 106 | 67 | 34 | 100 | 132 | P | 28 | -71 | -2.8 (1.41) | -1.3 (0.86) |
| Cutler 1992^25^ | US | 744 | 43 | 29 | 82 | 125 | P | 540 | -44 | -1.7 (0.59) | -0.9 (0.42) |
| Fotherby 1993^26^ | UK | 17 | 73 (66-79) | 78 | 100 | 179 | X | 35 | -79 | -8.0 (3.77) | 0.0 (2.39) |
| Nestel 1993^27^ | Australia | 66 | 66 | 45 | 100 | 146 | P | 42 | -84 | -3.9 (2.94) | -1.5 (2.25) |
| Redon-Mas 1993^28^ | Spain | 418 | 55 | 46 | NR | 163 | P | 28 | -109 | 0.9 (1.41) | 1.8 (0.92) |
| Howe 1994^29^ | Australia | 56 | 55 | 45 | NR | 145 | P | 42 | -78 | -4.2 (2.09) | -1.5 (2.14) |
| Schorr 1996^30^ | Germany | 16 | 64 | 56 | 100 | 134 | X | 28 | -71 | -7.2 (4.90) | -2.9 (2.61) |
| Cappuccio 1997^31^ | UK | 47 | 66.8 (60-78) | 49 | 89 | 163 | X | 30 | -83 | -7.2 (3.02) | -3.2 (1.42) |
| Cutler 1997^32^ | US | 1190 | 44 | 33 | 80 | 127 | P | 1095 | -40 | -1.2 (0.50) | -0.7 (0.40) |
| McCarron 1997^33^ | US | 99 | 52 | 42 | 73 | 139 | X | 28 | -55 | -4.9 (1.87) | -2.9 (1.10) |
| Meland 1997^34^ | Norway | 16 | 50 (20-69) | 19 | 100 | 145 | X | 56 | -66 | -4.0 (1.97) | -2.0 (1.25) |
| Yamamoto 1997^35^ | Japan | 36 | 53.3 (40-69) | 81 | 0 | 148 | P | 49 | -32 | -5.5 (6.40) | -3.3 (4.71) |
| Wing 1998^36^ | Australia | 17 | 61 (37-74) | 18 | NR | 160 | X | 42 | -59 | -7.0 (2.40) | -4.0 (1.37) |
| Ames 2001^37^ | US | 21 | 60 | 52 | 62 | 154 | X | 28 | -126 | -6.0 (3.33) | -2.3 (1.71) |
| Appel 2001^38^ | US | 681 | 66 | 47 | 76 | 128 | P | 90 | -40 | -4.3 (0.89) | -2.0 (0.61) |
| Akita 2003^39^ | US | 375 | 48 | 57 | 39 | 129 | X | 30 | -79 | -5.0 (1.27) | -2.0 (0.51) |
| Nowson 2003^40^ | Australia | 92 | 45 | 63 | NR | 118 | X | 28 | -88 | 0.4 (1.20) | 0.0 (1.00) |
| Berge-Landry 2004^41^ | US | 48 | 51 | 21 | 71 | 144 | X | 28 | -285 | -16.0 (4.56) | -8.0 (2.28) |
| Gates 2004^42^ | US | 12 | 64 | 50 | 100 | 144 | X | 28 | -89 | -7.0 (2.90) | -1.0 (1.83) |
| Swift 2005^43^ | UK | 40 | 50 | 58 | 0 | 159 | X | 28 | -78 | -8.0 (2.06) | -3.0 (1.11) |
| Cappuccio 2006^44^ | Ghana | 1013 | 55 | 62 | 0 | 128 | P | 180 | 6 | -2.5 (2.04) | -4.0 (1.61) |
| Melander 2007^45^ | Sweden | 39 | 53 | 49 | 100 | 132 | X | 28 | -89 | -6.5 (1.50) | -3.3 (1.20) |
| He 2009^46^ | UK | 169 | 50 | 33 | 42 | 146 | X | 42 | -55 | -4.8 (0.82) | -2.2 (0.43) |
| Meland 2009^47^ | Norway | 46 | 56 (20-75) | 26 | NR | 128 | P | 56 | -38 | -5.0 (2.73) | -5.0 (1.49) |
| Weir 2010^48^ | USA | 115 | 52 | 45 | 86 | 134 | X | 28 | -123 | -9.4 (0.99) | -5.7 (0.64) |
| Gijsbers 2015^49^ | Netherlands | 36 | 66 | 33 | NR | 137 | X | 28 | -98 | -7.5 (1.50) | -2.7 (0.76) |
| He 2015^50^ | China | 553 | 44 | 52 | 0 | 126 | P | 105 | -50 | -2.3 (1.16) | -0.9 (0.94) |
| Markota 2015^51^ | Bosnia and Herzegovina | 150 | 59 | 51 | NR | 175 | P | 60 | -28 | -4.9 (1.97) | -2.0 (0.60) |
| Riphagen 2016^52^ | Netherlands | 35 | 66 | 34 | 100 | 137 | X | 28 | -99 | -8.0 (1.50) | -2.9 (0.79) |
| Suckling 2016^53^ | UK | 46 | 58 | 48 | 70 | 136 | X | 42 | -49 | -4.3 (1.95) | -1.6 (1.15) |
| Parvanova 2018^54^ | Italy | 115 | 64 | 11 | NR | 146 | P | 90 | -44 | -4.7 (2.02) | -3.0 (1.07) |

**References**

1. Parijs J, Joossens J, Linden L, Verstreken G, Amery A. Moderate sodium restriction and diuretics in the treatment of hypertension. *Am Heart J.* 1973;85(1):22-34.

2. MacGregor G, Markandu N, Best F, Elder D, Cam J, Sagnella G, et al. Double-blind randomized crossover trial of moderate sodium restriction in essential hypertension. *Lancet.* 1982;1(8268):351-355.

3. Puska P, Iacono J, Nissinen A, Korhonen H, Vartianinen E, Pietinen P, et al. Controlled, randomized trial of the effect of dietary fat on blood pressure. *Lancet.* 1983;1(8314-5):1-5.

4. Silman AJ, Mitchell P, Locke C, Humpherson P. Evaluation of the effectiveness of a low sodium diet in the treatment of mild to moderate hypertension. *Lancet.* 1983;1(8335):1179-1182.

5. Watt GCM, Edwards C, Hart JT. Dietary sodium restriction for mild hypertension in general practice. *BMJ.* 1983;286(6363):432-436.

6. Erwteman TM, Nagelkerke N, Lubsen J, Koster M, Dunning AJ. beta Blockade, diuretics, and salt restriction for the management of mild hypertension: A randomized double blind trial. *BMJ.* 1984;289(6442):406-409.

7. Gillies A, Carney S, Smith A, Waga S. Adjunctive effect of salt restriction on antihypertensive efficacy. *Clin Exp Pharmacol Physiol.* 1984;11(4):395-398.

8. Richards A, Espiner E, Maslowski A, Nicholls M, Ikram H, Hamilton E, et al. Blood pressure response to moderate sodium restriction and to potassium supplementation in mild essential hypertension. *Lancet.* 1984;I(8380):757-761. <http://onlinelibrary.wiley.com/o/cochrane/clcentral/articles/469/CN-01370469/frame.html>.

9. Grobbee D, Hofman A, Roelandt J, Boomsma F, Schalekamp M, Valkenburg H. Sodium restriction and potassium supplementation in young people with mildly elevated blood pressure. *J Hypetens.* 1987;5(1):115-119.

10. MacGregor GA, Markandu ND, Singer DRJ. Moderate sodium restriction with angiotensin converting enzyme inhibitor in essential hypertension: A double blind study. *BMJ.* 1987;294(6571):531-534.

11. Morgan T, Anderson A. Sodium restriction can delay the return of hypertension in patients previously well-controlled on drug therapy [Clinical Trial; Comparative Study; Controlled Clinical Trial; Research Support, Non-U.S. Gov't]. *Can J Physiol Pharmacol.* 1987;65(8):1752-1755. <http://onlinelibrary.wiley.com/o/cochrane/clcentral/articles/339/CN-00051339/frame.html>.

12. Nowson CA, Morgan TO. Change in blood pressure in relation to change in nutrients effected by manipulation of dietary sodium and potassium. *Clin Exp Pharmacol Physiol.* 1988;15(3):225-242.

13. Staessen J, Bulpitt C, Fagard R, Joossens J, Lijnen P, Amery A. Salt intake and blood pressure in the general population: a controlled intervention trial in two towns. *J Hypetens.* 1988;6(12):965-973.

14. Chalmers JP, Doyle AE, Hopper JL, Howe PRC, Matthews PG, Mathews J, et al. Effects of replacing sodium intake in subjects on a low sodium diet: A crossover study. *Clin Exp Hypertens A.* 1989;11(5-6):1011-1024.

15. Chalmers JP, Doyle AE, Hopper JL, Howe PRC, Matthews PG, Mathews J, et al. Fall in blood pressure with modest reduction in dietary salt intake in mild hypertension. *Lancet.* 1989;1(8635):399-402.

16. Dodson PM, Beevers M, Hallworth R, Webberley MJ, Fletcher RF, Taylor KG. Sodium restriction and blood pressure in hypertensive type II diabetics: Randomized blind controlled and crossover studies of moderate sodium restriction and sodium supplementation. *BMJ.* 1989;298(6668):227-230.

17. MacGregor GA, Markandu ND, Sagnella GA, Singer DRJ, Cappuccio FP. Double-blind study of three sodium intakes and long-term effects of sodium restriction in essential hypertension. *Lancet.* 1989;2(8674):1244-1247.

18. Parker M, Puddey IB, Beilin LJ, Vandongen R. Two-way factorial study of alcohol and salt restriction in treated hypertensive men. *Hypertension.* 1990;16(4):398-406.

19. Carney S, Gillies A, Smith A, Smitham S. Increased dietary sodium chloride in patients treated with antihypertensive drugs. *Clin Exp Hypertens A.* 1991;13(3):401-407.

20. Singer DRJ, Markandu ND, Sugden AL, Miller MA, MacGregor GA. Sodium restriction in hypertensive patients treated with a converting enzyme inhibitor and a thiazide. *Hypertension.* 1991;17(6 I):798-803.

21. Alli C, Avanzini F, Bettelli G, Bonati M, Colombo F, Corso R, et al. Feasibility of a long-term low-sodium diet in mild hypertension. *J Hum Hypertens.* 1992;6(4):281-286.

22. Arroll B. The Auckland blood pressure control study: a randomized controlled trial of physical activity and salt restriction in persons. *Medical and health sciences.* 1992;PhD. <http://onlinelibrary.wiley.com/o/cochrane/clcentral/articles/166/CN-00796166/frame.html>.

23. Benetos A, Yang-Yan X, Cuche JL, Hannaert P, Safar M. Arterial effects of salt restriction in hypertensive patients. A 9-week, randomized, double-blind, crossover study. *J Hypetens.* 1992;10(4):355-360.

24. Cobiac L, Nestel PJ, Wing LMH, Howe PRC. A low-sodium diet supplemented with fish oil lowers blood pressure in the elderly. *J Hypetens.* 1992;10(1):87-92.

25. Cutler JA, Whelton PK, Appel L, Charleston J, Dalcin AT, Ewart C, et al. The effects of nonpharmacologic interventions on blood pressure of persons with high normal levels: Results of the trials of hypertension prevention, phase I. *JAMA.* 1992;267(9):1213-1220.

26. Fotherby MD, Potter JF. Effects of moderate sodium restriction on clinic and twenty-four-hour ambulatory blood pressure in elderly hypertensive subjects. *J Hypetens.* 1993;11(6):657-663.

27. Nestel PJ, Clifton PM, Noakes M, McArthur R, Howe PR. Enhanced blood pressure response to dietary salt in elderly women, especially those with small waist: Hip ratio. *J Hypetens.* 1993;11(12):1387-1394.

28. Redon-Mas J, Abellan-Aleman J, Aranda-Lara P, de la Figuera-von Wichmann M, Luque-Otero M, Rodicio-Diaz JL, et al. Antihypertensive activity of verapamil: Impact of dietary sodium. *J Hypetens.* 1993;11(6):665-671.

29. Howe PRC, Lungershausen YK, Cobiac L, Dandy G, Nestel PJ. Effect of sodium restriction and fish oil supplementation on BP and thrombotic risk factors in patients treated with ACE inhibitors. *J Hum Hypertens.* 1994;8(1):43-49.

30. Schorr U, Distler A, Sharma AM. Effect of sodium chloride- and sodium bicarbonate-rich mineral water on blood pressure and metabolic parameters in elderly normotensive individuals: A randomized double-blind crossover trial. *J Hypetens.* 1996;14(1):131-135.

31. Cappuccio FP, Markandu ND, Carney C, Sagnella GA, MacGregor GA. Double-blind randomized trial of modest salt restriction in older people. *Lancet.* 1997;350(9081):850-854.

32. Cutler JA. Effects of weight loss and sodium reduction intervention on blood pressure and hypertension incidence in overweight people with high-normal blood pressure: The trials of hypertension prevention, phase II. *Arch Intern Med.* 1997;157(6):657-667.

33. McCarron DA, Weder AB, Egan BM, Krishna GG, Morris CD, Cohen M, et al. Blood pressure and metabolic responses to moderate sodium restriction in isradipine-treated hypertensive patients. *Am J Hypertens.* 1997;10(1):68-76.

34. Meland E, Laerum E, Aakvaag A, Ulvik R, Høstmark A. Salt restriction: effects on lipids and insulin production in hypertensive patients [Clinical Trial; Randomized Controlled Trial; Research Support, Non-U.S. Gov't]. *Scand J Clin Lab Invest.* 1997;57(6):501-505. <http://onlinelibrary.wiley.com/o/cochrane/clcentral/articles/677/CN-00144677/frame.html>.

35. Yamamoto H. Randomized controlled trial of salt-restriction program for primary prevention of hypertension in the community. *Journal of the osaka city medical center.* 1997;46(3-4):255-267. <http://onlinelibrary.wiley.com/o/cochrane/clcentral/articles/180/CN-00766180/frame.html>.

36. Wing L, Arnolda L, Harvey P, Upton J, Molloy D, Gabb G. Low-dose diuretic and/or dietary sodium restrication when blood pressure is resistant to ACE inhibitor. *Blood pressure.* 1998;7(5-6):299-307. <http://onlinelibrary.wiley.com/o/cochrane/clcentral/articles/529/CN-00253529/frame.html>.

37. Ames RP. The effect of sodium supplementation on glucose tolerance and insulin concentrations in patients with hypertension and diabetes mellitus. *Am J Hypertens.* 2001;14(7 I):653-659.

38. Appel L, Espeland M, Easter L, Wilson A, Folmar S, Lacy C. Effects of reduced sodium intake on hypertension control in older individuals: results from the Trial of Nonpharmacologic Interventions in the Elderly (TONE) [Clinical Trial; Randomized Controlled Trial; Research Support, U.S. Gov't, P.H.S.]. *Arch Intern Med.* 2001;161(5):685-693. <http://onlinelibrary.wiley.com/o/cochrane/clcentral/articles/462/CN-00327462/frame.html>

<https://jamanetwork.com/journals/jamainternalmedicine/articlepdf/647555/ioi00286.pdf>.

39. Akita S, Sacks F, Svetkey L, Conlin P, Kimura G. Effects of the Dietary Approaches to Stop Hypertension (DASH) diet on the pressure-natriuresis relationship [Clinical Trial; Multicenter Study; Randomized Controlled Trial]. *Hypertension.* 2003;42(1):8-13. doi:10.1161/01.HYP.0000074668.08704.6E.

40. Nowson CA, Morgan TO, Gibbons C. Decreasing Dietary Sodium while Following a Self-Selected Potassium-Rich Diet Reduces Blood Pressure. *J Nutr.* 2003;133(12):4118-4123.

41. Berge-Landry H, James G. Serum electrolyte, serum protein, serum fat and renal responses to a dietary sodium challenge: allostasis and allostatic load [Clinical Trial; Randomized Controlled Trial; Research Support, U.S. Gov't, P.H.S.]. *Ann Hum Biol.* 2004;31(4):477-487. doi:10.1080/03014460412331281746.

42. Gates P, Tanaka H, Hiatt W, Seals D. Dietary sodium restriction rapidly improves large elastic artery compliance in older adults with systolic hypertension [Clinical Trial; Randomized Controlled Trial; Research Support, Non-U.S. Gov't; Research Support, U.S. Gov't, P.H.S.]. *Hypertension.* 2004;44(1):35-41. doi:10.1161/01.HYP.0000132767.74476.64.

43. Swift PA, Markandu ND, Sagnella GA, He FJ, MacGregor GA. Modest salt reduction reduces blood pressure and urine protein excretion in black hypertensives: A randomized control trial. *Hypertension.* 2005;46(2):308-312.

44. Cappuccio F, Kerry S, Micah F, Plange-Rhule J, Eastwood J. A community programme to reduce salt intake and blood pressure in Ghana [Randomized Controlled Trial; Research Support, Non-U.S. Gov't]. *BMC Public Health.* 2006;6:13. doi:10.1186/1471-2458-6-13.

45. Melander O, Wowern F, Frandsen E, Burri P, Willsteen G, Aurell M, et al. Moderate salt restriction effectively lowers blood pressure and degree of salt sensitivity is related to baseline concentration of renin and N-terminal atrial natriuretic peptide in plasma. *J Hypetens.* 2007;25(3):619-627.

46. He F, Marciniak M, Visagie E, Markandu N, Anand V, Dalton R, et al. Effect of modest salt reduction on blood pressure, urinary albumin, and pulse wave velocity in white, black, and Asian mild hypertensives. *Hypertension.* 2009;54(3):482-488.

47. Meland E, Aamland A. Salt restriction among hypertensive patients: modest blood pressure effect and no adverse effects [Randomized Controlled Trial; Research Support, Non-U.S. Gov't]. *Scand J Prim Health Care.* 2009;27(2):97-103. doi:10.1080/02813430802661795.

48. Weir MR, Yadao AM, Purkayastha D, Charney AN. Effects of high- and low-sodium diets on ambulatory blood pressure in patients with hypertension receiving aliskiren. *J Cardiovasc Pharmacol Ther.* 2010;15(4):356-363.

49. Gijsbers L, Dower JI, Schalkwijk CG, Kusters YHAM, Bakker SJL, Hollman PCH, et al. Effects of sodium and potassium supplementation on endothelial function: A fully controlled dietary intervention study. *Br J Nutr.* 2015;114(9):1419-1426.

50. He FJ, Wu Y, Feng XX, Ma J, Ma Y, Wang H, et al. School based education programme to reduce salt intake in children and their families (School-EduSalt): Cluster randomized controlled trial. *BMJ.* 2015;350:h770.

51. Pinjuh MN, Rumboldt M, Rumboldt Z. Emphasized warning reduces salt intake: A randomized controlled trial [Journal: Article]. *J Am Soc Hypertens.* 2015;9(3):214-220. doi:10.1016/j.jash.2014.12.022.

52. Riphagen IJ, Gijsbers L, van Gastel MD, Kema IP, Gansevoort RT, Navis G, et al. Effects of potassium supplementation on markers of osmoregulation and volume regulation: results of a fully controlled dietary intervention study. *J Hypetens.* 2016;34(2):215-220.

53. Suckling RJ, He FJ, Markandu ND, Macgregor GA. Modest salt reduction lowers blood pressure and albumin excretion in impaired glucose tolerance and type 2 diabetes mellitus: A randomized double-blind trial. *Hypertension.* 2016;67(6):1189-1195.

54. Parvanova A, Trillini M, Podesta MA, Iliev IP, Ruggiero B, Abbate M, et al. Moderate salt restriction with or without paricalcitol in type 2 diabetes and losartan-resistant macroalbuminuria (PROCEED): a randomized, double-blind, placebo-controlled, crossover trial. *Lancet Diabetes Endocrinol.* 2018;6(1):27-40.

# Supplementary Appendix 2 Data of salt substitute trials

| **Study** | **Study country** | **No. of participants** | **Mean (range) age (years)** | **Female (%)** | **Baseline SBP** | **Design** | **Study duration** | **Change in UNa (mmol/24-hour)** | **Change in UK (mmol/24-hour)** | **Difference in change of SBP (mmHg)  mean (SE)** |
| --- | --- | --- | --- | --- | --- | --- | --- | --- | --- | --- |
| Barros 2015^1^ | Brazil | 35 | 55.5 | 65.7 | 143.17 | P | 28 days | -47.81 | -23.98 | -9.70 (7.89) |
| Gilleran 1996^2^ | UK | 40 | 63 | 40 | 166 | P | 3 months | 13.70 | 18.70 | -6.90 (9.55) |
| Omvik 1995^3^ | Norway | 40 | 44 | 32 | 156 | P | 6 months | -1.00 | 24.00 | -3.00 (9.23) |
| Sarkkinen 2011^4^ | Finland | 45 | 56 | 49 | 140 | P | 8 weeks | -29.00 | 32.00 | -12.00 (4.12) |
| Suppa 1988^5^ | Italy | 322 | 47 | 37 | 150 | P | 4 weeks | 6.80 | 14.10 | -2.50 (3.06) |
| ^1*^Yang (a) 2018^6^ | China | 51 | 67 | 59 | 159 | P | 6 months | -54.00 | 8.10 | -10.00 (3.88) |
| ^2*^Yang (b) 2018^6^ | China | 75 | 66 | 56 | 158 | P | 6 months | -76.00 | 10.30 | -6.00 (4.39) |
| ^3*^Zhou (a) 2009^7^ | China | 126 | 67 | 57 | 159 | P | 6 months | -68.00 | 8.30 | -11.80 (2.98) |
| ^4*^Zhou (b) 2009^7^ | China | 122 | 67 | 53 | 124 | P | 6 months | -67.00 | 11.00 | -7.00 (1.92) |
| Bernabe-Ortiz 2020^8^ | Peru | 2376 | 42 | 50 | 113 | Stepped-wedge | 30 months | 0.43 | 27.39 | -1.29 (0.45) |
| Yu 2021^9^ | India | 502 | 62 | 59 | 133 | P | 3 months | -3.04 | 6.14 | -4.58 (0.82) |

^1*^Patients with isolated systolic hypertension

^2*^Patients with nonisolated systolic hypertension

^3*^Patients with hypertension

^4*^Patients with normal blood pressure

References

1. Barros CL, Sousa AL, Chinem BM, Rodrigues RB, Jardim TS, Carneiro SB, et al. Impact of light salt substitution for regular salt on blood pressure of hypertensive patients. *Arq Bras Cardiol.* 2015;104(2):128-135.

2. Gilleran G, O'Leary M, Bartlett WA, Vinall H, Jones AF, Dodson PM. Effects of dietary sodium substitution with potassium and magnesium in hypertensive type II diabetics: a randomized blind controlled parallel study. *J Hum Hypertens.* 1996;10(8):517-521.

3. Omvik P, Myking OL. Unchanged central hemodynamics after six months of moderate sodium restriction with or without potassium supplement in essential hypertension. *Blood Press.* 1995;4(1):32-41.

4. Sarkkinen ES, Kastarinen MJ, Niskanen TH, Karjalainen PH, Venalainen TM, Udani JK, et al. Feasibility and antihypertensive effect of replacing regular salt with mineral salt -rich in magnesium and potassium- in subjects with mildly elevated blood pressure. *Nutr J.* 2011;10:88.

5. Suppa G, Pollavini G, Alberti D, Savonitto S. Effects of a low-sodium high-potassium salt in hypertensive patients treated with metoprolol: a multicentre study. *J Hypertens.* 1988;6(10):787-790.

6. Yang GH, Zhou X, Ji WJ, Liu JX, Sun J, Shi R, et al. Effects of a low salt diet on isolated systolic hypertension: A community-based population study. *Medicine (Baltimore).* 2018;97(14):e0342.

7. Zhou X, Liu JX, Shi R, Yang N, Song DL, Pang W, et al. Compound ion salt, a novel low-sodium salt substitute: from animal study to community-based population trial. *Am J Hypertens.* 2009;22(9):934-942.

8. Bernabe-Ortiz A, Sal YRVG, Ponce-Lucero V, Cardenas MK, Carrillo-Larco RM, Diez-Canseco F, et al. Effect of salt substitution on community-wide blood pressure and hypertension incidence. *Nat Med.* 2020;26(3):374-378.

9. Yu J, Thout SR, Li Q, Tian M, Marklund M, Arnott C, et al. Effects of a reduced-sodium added-potassium salt substitute on blood pressure in rural Indian hypertensive patients: a randomized, double-blind, controlled trial. *Am J Clin Nutr.* 2021;114(1):185-193.

# Supplementary Appendix 3 Participants’ characteristics of cohort data in SSaSS

|  | **Group** | | | |
| --- | --- | --- | --- | --- |
| **Characteristic** | **Overall,**  **N = 449^1^** | **Control,**  **N = 207^1^** | **Intervention,**  **N = 242^1^** | **p-value^2^** |
| Female | 239 (53%) | 107 (52%) | 132 (55%) | 0.610 |
| Age, yrs | 65 (7) | 66 (7) | 65 (7) | 0.133 |
| Systolic blood pressure, mmHg | 162 (24) | 161 (25) | 162 (23) | 0.859 |
| Diastolic blood pressure, mmHg | 93 (14) | 93 (14) | 93 (14) | 0.832 |
| 24-h urinary sodium, mmol | 191 (79) | 186 (76) | 195 (81) | 0.214 |
| 24-h urinary potassium, mmol | 37 (16) | 37 (15) | 36 (16) | 0.798 |
| Change of systolic blood pressure from baseline to follow up, mmHg | -18 (28) | -17 (25) | -18 (30) | 0.585 |
| Change of diastolic blood pressure from baseline to follow up, mmHg | -10 (16) | -9 (15) | -11(17) | 0.225 |
| Change of 24-h urinary sodium from baseline to follow up, mmol | -30 (94) | -23 (96) | -37 (93) | 0.129 |
| Change of 24-h urinary potassium from baseline to follow up, mmol | 16 (27) | 4 (19) | 26 (30) | <0.001 |
| Height, cm | 160 (8) | 160 (8) | 160 (8) | 0.808 |
| Weight, kg | 66 (10) | 66 (10) | 66 (11) | 0.890 |
| BMI, kg/m^2^ | 25.6 (3.4) | 25.5 (3.2) | 25.6 (3.6) | 0.970 |
| Past smoker | 136 (30%) | 63 (30%) | 73 (30%) | >0.999 |
| Current smoker | 80 (18%) | 32 (15%) | 48 (20%) | 0.278 |
| Education |  |  |  | 0.868 |
| College | 3 (0.7%) | 2 (1.0%) | 1 (0.4%) |  |
| Senior high school or technical secondary school | 20 (4.5%) | 10 (4.8%) | 10 (4.1%) |  |
| Junior high school | 77 (17%) | 34 (16%) | 43 (18%) |  |
| Primary school or lower | 349 (78%) | 161 (78%) | 188 (78%) |  |
| ^1^n (%); Mean (SD) | | | | |
| ^2^Pearson’s Chi-squared test; One-way ANOVA; Fisher’s exact test | | | | |

# Supplementary Appendix 4 Linear mixed models fitted using SSaSS individual data

| **Model** | **Dependent variable** | **Fixed effects** | **Random effect** |
| --- | --- | --- | --- |
| Model 1 | Change in SBP from baseline to follow-up | - Change in 24-hour urinary sodium from baseline to follow-up - Change in potassium from baseline to follow-up | Cluster (village) |
| Model 2 | Change in SBP from baseline to follow-up | - Change in 24-hour urinary sodium from baseline to follow-up - Change in potassium from baseline to follow-up - Sex - Baseline age - Baseline SBP | Cluster (village) |
| Model 3 | Change in SBP from baseline to follow-up | - Change in 24-hour urinary sodium from baseline to follow-up - Change in potassium from baseline to follow-up - Sex - Baseline age - Baseline SBP - Baseline sodium - Baseline potassium | Cluster (village) |
| Model 4 | Change in SBP from baseline to follow-up | - Change in 24-hour urinary sodium from baseline to follow-up - Change in potassium from baseline to follow-up - Interaction between change in sodium and change in potassium - Sex - Baseline age - Baseline SBP - Baseline sodium - Baseline potassium | Cluster (village) |
| Model 5 | Change in SBP from baseline to follow-up | - Change in 24-hour urinary sodium from baseline to follow-up - Change in potassium from baseline to follow-up - Sex - Baseline age - Baseline SBP - Baseline sodium - Baseline potassium - Interaction between change in sodium and baseline potassium - Interaction between change in potassium and baseline sodium | Cluster (village) |

# Supplementary Figure 1 Observed systolic blood pressure reduction in the Salt Substitute and Stoke Study (dark grey) and expected effects of sodium reduction (black) and potassium increase (white) on systolic blood pressure for different data sources and statistical models assuming 95% of dietary sodium and 80% of dietary potassium were excreted through urine


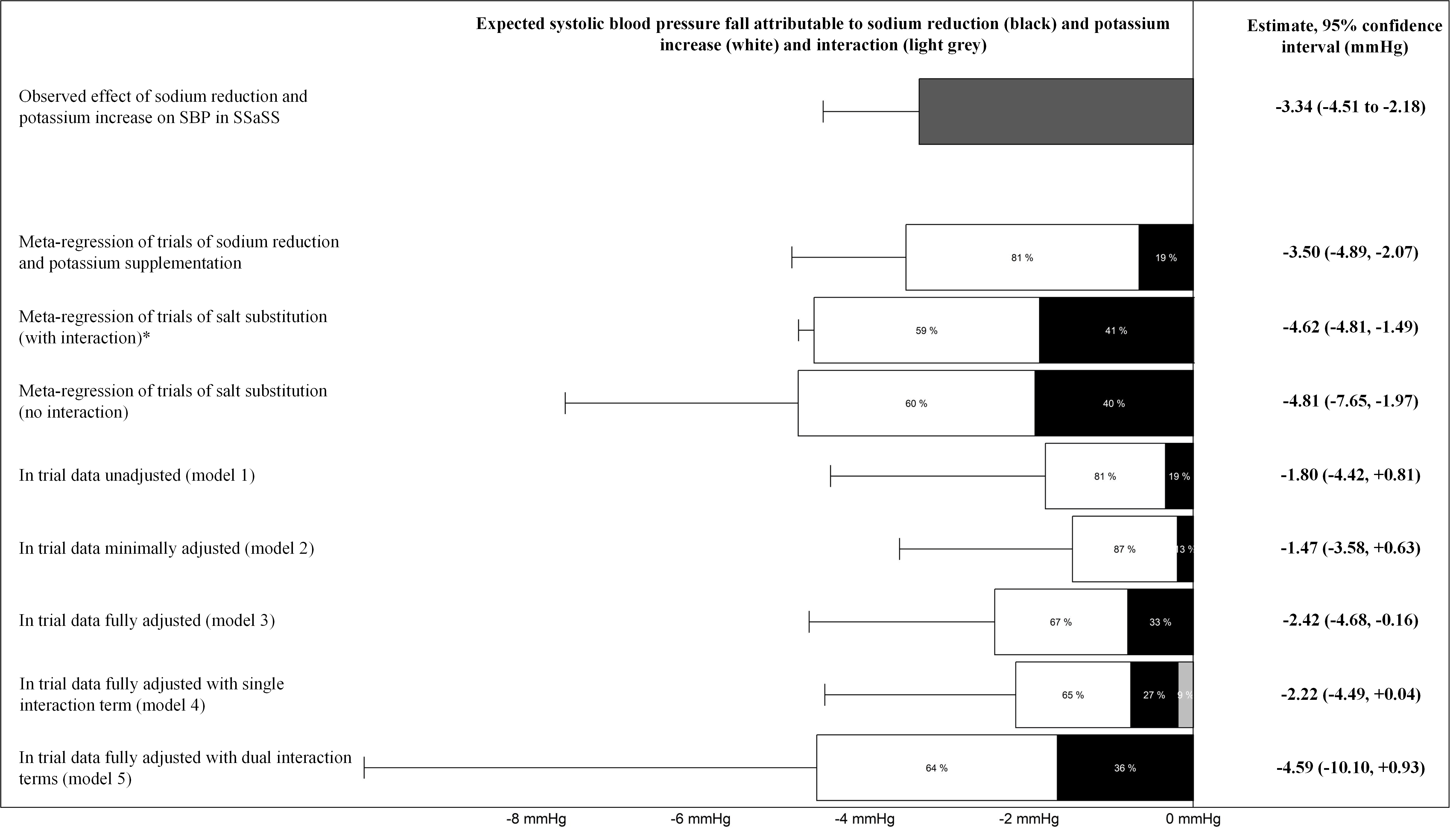


* for this model, the interaction term was an 0.90 mmHg rise in SBP attributable to the joint effects of sodium reduction and potassium supplementation, which is not shown in the figure.

# Supplementary Figure 2 Observed systolic blood pressure reduction in the Salt Substitute and Stoke Study (dark grey) and expected effects of sodium reduction (black) and potassium increase (white) on systolic blood pressure for different data sources and statistical models with no adjustment to dietary intake of sodium and potassium


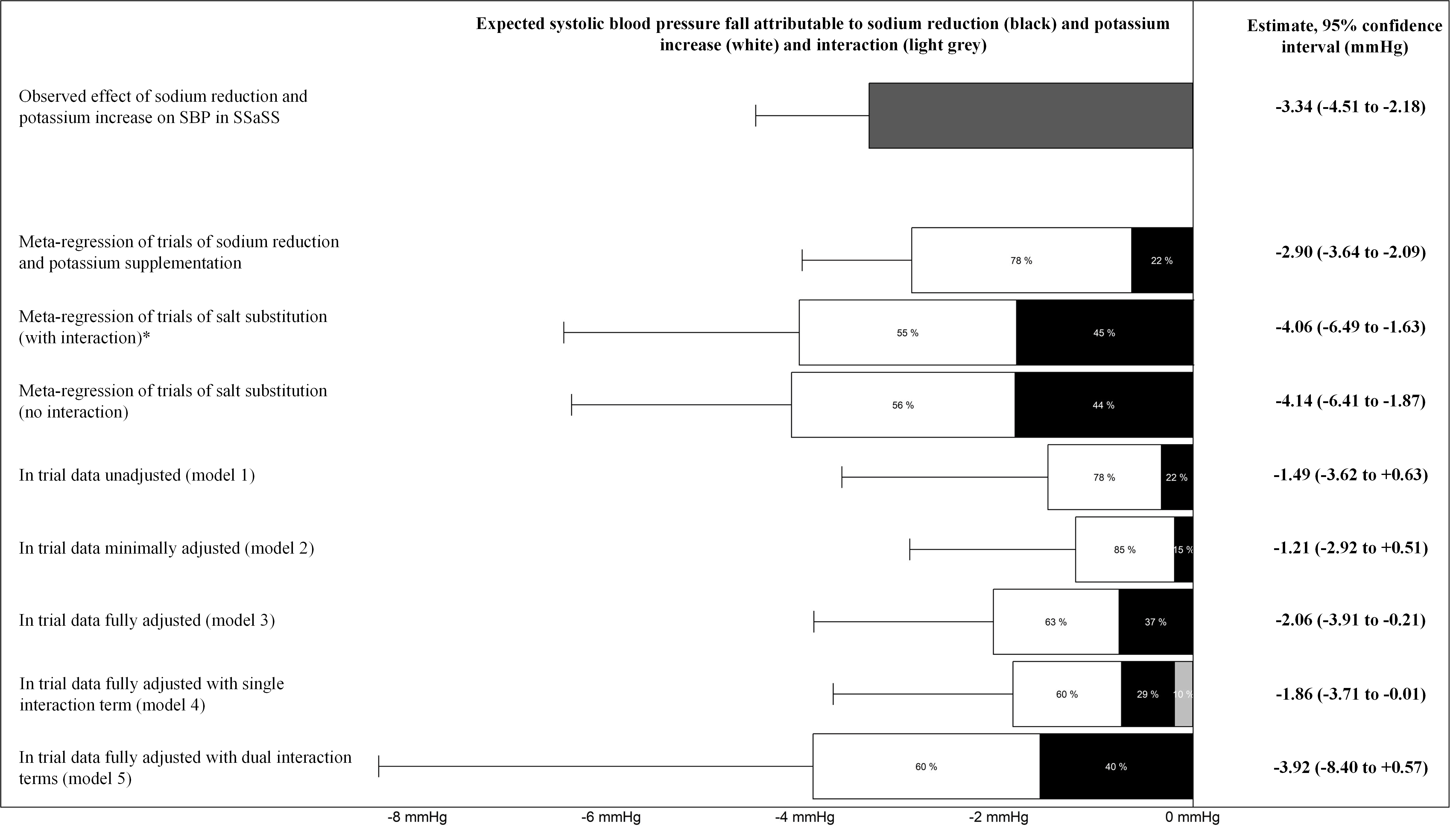


* for this model, the interaction term was an 0.74 mmHg rise in SBP attributable to the joint effects of sodium reduction and potassium supplementation, which is not shown in the figure.
